# Analysis of the quality of hospital information systems audit trails

**DOI:** 10.1186/1472-6947-13-84

**Published:** 2013-08-06

**Authors:** Ricardo Cruz-Correia, Isabel Boldt, Luís Lapão, Cátia Santos-Pereira, Pedro Pereira Rodrigues, Ana Margarida Ferreira, Alberto Freitas

**Affiliations:** 1 CINTESIS – Centre for Research in Health Technologies and Information Systems, Faculdade de Medicina da Universidade do Porto, Porto, Portugal; 2 International Public Health and Biostatistics and WHO Collaborating Center for Health Workforce Policy and Planning, Instituto de Higiene e Medicina Tropical – Universidade Nova de Lisboa, Lisbon, Portugal; 3 Department of Health Information and Decision Sciences, Faculdade de Medicina da Universidade do Porto, Porto, Portugal

## Abstract

**Background:**

Audit Trails (AT) are fundamental to information security in order to guarantee access traceability but can also be used to improve Health information System’s (HIS) quality namely to assess how they are used or misused. This paper aims at analysing the existence and quality of AT, describing scenarios in hospitals and making some recommendations to improve the quality of information.

**Methods:**

The responsibles of HIS for eight Portuguese hospitals were contacted in order to arrange an interview about the importance of AT and to collect audit trail data from their HIS. Five institutions agreed to participate in this study; four of them accepted to be interviewed, and four sent AT data. The interviews were performed in 2011 and audit trail data sent in 2011 and 2012. Each AT was evaluated and compared in relation to data quality standards, namely for completeness, comprehensibility, traceability among others. Only one of the AT had enough information for us to apply a consistency evaluation by modelling user behaviour.

**Results:**

The interviewees in these hospitals only knew a few AT (average of 1 AT per hospital in an estimate of 21 existing HIS), although they all recognize some advantages of analysing AT. Four hospitals sent a total of 7 AT – 2 from Radiology Information System (RIS), 2 from Picture Archiving and Communication System (PACS), 3 from Patient Records. Three of the AT were understandable and three of the AT were complete. The AT from the patient records are better structured and more complete than the RIS/PACS.

**Conclusions:**

Existing AT do not have enough quality to guarantee traceability or be used in HIS improvement. Its quality reflects the importance given to them by the CIO of healthcare institutions. Existing standards (e.g. ASTM:E2147, ISO/TS 18308:2004, ISO/IEC 27001:2006) are still not broadly used in Portugal.

## Background

The practice of medicine has been described as being dominated by how well information is collected, processed, retrieved, and communicated [1]. An important challenge is to guarantee the good working conditions for health professionals to access clinical data while Hospital Information Systems (HIS) are still being developed [2]. Although great advances have been made over the years, on-demand access to clinical information is still inadequate in many settings, contributing to duplication of effort, excess costs, adverse events, and reduced efficiency [3]. Shapiro et al. [4] found that, although doctors from emergency department believe their patients would benefit from the use of longitudinal records, they only try to obtain such data in 10% of the cases. Furthermore, Hripcsak et al. [5] described access rates to Clinical information System (WebCIS) in the emergency department [5], indicating that data generated before the current emergency visit are accessed often, but by no means in a majority of times (only 5% to 20% of the encounters), even when the user was notified of the availability of such data. Cruz-Correia et al. [6] have shown that not many clinical reports are still used one year after creation, regardless of the context in which they were created, although significant differences existed in reports created during distinct hospital encounter types. Also, the usage of patients’ past information (data from previous encounters) varied according to the setting of healthcare and content. This type of research is only possible when records with retention requirements that show who has accessed what in a HIS, when and what operations were involved are properly collected. These records are called Audit Trails (AT) [7]. Although, AT are generally just intended to trace user events when an incident occurs, they can also be used to improve HIS. Some attention as already been given define the requirements of AT in healthcare, namely by RFC 3881, IHE-ATNA, DICOM. These standards are being referenced in some of the most important projects today (e.g. epSOS or “Meaningful use stage 2” proposed rule).

### Aim

This paper aims to address the issues related to the existence and quality of AT regarding patient records, to describe the Hospital’s scenario and to produce recommendations. It comprises studies on:

•the opinions of Hospital Chief Information Officer (CIO) regarding AT,

•the data elements to include in AT,

•quality of data dimensions to use in AT, and

•an analysis of the existing AT within Portuguese Hospitals.

### State of art

The most common AT function is the access management [8]. Nonetheless, other relevant functions exist such as the monitoring of employee behaviour and computer / information system failures. An AT can be used and analysed with many different purposes, although with a higher impact when used as evidence in healthcare practices lawsuits, or in the internal validation of new practices, like the introduction of a new IS. The best way to assess the accountability in lawsuits would be to backtrack the records to their state at the point of care; thus, a trail is mandatory [9]. On the clinical point of view, relevance has been given to the importance of AT, for example, in the validation of clinical decision support systems in paediatric critical care [10] or in the integration of multi-centric clinical trial data management [11].

Creating an AT implies the implementation of several audit controls [8], possibly integrated among multiple IS (e.g. Radiology Information System (RIS), Electronic Health Record (EHR) and Picture Archiving and Communication System (PACS)). RIS are one of the groups of systems where this integration has been better audited [12]. The resulting audit files, possibly distributed in the network, are what in computer science is commonly called “log files” of an IS. However, log files are most of the times lightly defined by software companies having only debugging purposes. The notion of AT extends that of a log file, in the sense that it can also be used to monitor the development of electronic health records, the import and export of protected health information from and to external entities, the modification, viewing and deletion of information, making it possible to rely on the integrity of the records in health information exchanges and the data fed into personal health records [8]. Different institutions as EuroRec [13], the ISO/HL7 21731 (RIM) [14] standard, and IHE [15] have approached this issue. Information Technology (IT) governance practices (e.g., IT Infrastructure Library (ITIL), which recommends on the use of AT for proper service level management) are being introduced in many Hospitals to cope with increasing levels of information quality and safety requirements. But the standard maturity levels of hospital IT departments is still not enough to reach the level of frequent use of AT [16].

In 2007, the Portuguese Government approved the Law 46/2007 that regulates the administrative documents access and reutilization. This law states that the patient is the owner of his/her health information and he/she has the right to request access to his/her medical information and to access it without any intermediary [17]. Unfortunately, there is no Portuguese law or regulation enforcing health care institutions to have complete and secure audit trails in their own institutions and the initiatives such as HIPAA, HITECH or EuroRec are still not considered when defining the security requirements for new Healthcare Information Systems.

The application of data mining and machine learning techniques to medical knowledge discovery tasks is now a growing research area. These techniques vary widely and are based on data-driven conceptualizations, model-based definitions or are a combination of data-based knowledge with human-expert knowledge [18]. Another important technique in this area is process mining. It aims at deriving process models from observed user behaviour [19], by extracting knowledge from the event logs recorded in the IS [20]. The AT can be used to uncover process, control, data, organizational, and social structures [21-23]. AT have some important interdependencies [24], namely with Policy (who is authorized, to access what), Operational Assurance, Identification and Authentication (user accountability), Logical Access Control (identify breakdowns, audit use of resources), Contingency Planning (reconstruction of the state of the system), Incident Response (to understand the magnitude of the incident), and Cryptography (to alert for modification of the audit trail).

## Methods

This section presents the methods used in the interview, and in the analysis of the AT.

### Participants in the interview and collection of AT

A convenience sample of CIOs was selected with the aim to find out about the AT usage in their institutions. Due to the sensitive nature of this information, each institution involved was made anonymous. The institutions were identified as Hospitals A, B, C, D, E, F, G and H, 4 District (DH) and 4 Central Hospitals, as shown in Table 1.

**Table 1:** CIOs of institutions interviewed and AT collected

	**A**	**B**	**C**	**D**	**E**	**F**	**G**	**H**
	**(DH)**	**(DH)**	**(CH)**	**(DH)**	**(CH)**	**(DH)**	**(CH)**	**(CH)**
Interview	✓	✓	✓		✓	Refused to answer		
AT Collection	✓	✓		✓	✓			

*DH*: –District Hospital, *CH:* –Central Hospital.

Three of them refused to participate in the study (F, G and H). In the other 5 institutions it was not possible to perform interviews and collect AT in all hospitals. In particular, we could not collect AT in Hospital C because the provider of the IS was an external company and did not allow the collection. We asked for samples of the existing AT, and then for the AT in the original format if they were flat files or tabular format exports (e.g. CSV) if they were in databases. The only modification we asked for was the anonimization regarding patients and system users.

### Interviews to hospital CIOs

Aiming to describe the current scenario in Portuguese Hospitals, interviews were performed to representatives of IT departments (CIOs). These representatives were the director of the department (n = 4) or someone that was directly responsible to maintain the HIS (n = 1). These semi-structured interviews were performed by telephone during January 2011, and apart from other interesting topics that could emerge during the interview, at least the following questions were raised in the following order:

1. What is the number of IS that have AT?

2. What is the frequency that someone asks to use this data?

3. What were the main reasons to access AT?

4. What are the potential main benefits to record these AT?

5. What are the main problems to record these AT?

6. Have you direct access to them, or need to ask it from the software providers?”

### Data elements important for the completeness of AT and quality of data dimensions applicable to AT

Based on the work of Halanka et al. described in [25], to be a quality AT it should have at least the following data elements: 1) Time; 2) Date; 3) Information Accessed and 4) User ID. Other studies [25-27] and a ASTM standard specification [28] also describe as being important to include other elements like 1) User Position/Role; 2) Patient Location (e.g. Ward); 3) Actualization Reason; 4) Chart Access Reason; 5) Document Code; 6) Orders Entered; 7) Service/Department and 8) User’s Place of Work (e.g. Ward). According to the CCHIT (Certification Commission for Healthcare Information Technology) the following elements are also mandatory:1) date and time of the event, 2) the component of the information system (e.g., software component, hardware component) where the event occurred, 3) type of event (including: data description and patient identifier when relevant), 4) subject identity (e.g. user identity); and 5) the outcome (success or failure) of the event. Table 2 presents the data elements considered important by each of these studies, compares them with the ones needed for our own process-mining studies [29], and presents an overall importance rate based on the frequency each element is referred.

**Table 2:** Mandatory fields analyzed in other AT studies and by ourselves in this study

	**RFC 3881**[41]	**Halamka**[25]	**Røstad**[26]	**Zhang**[27]	**E2147**[28]	**Needed for our process-mining studies**[29]	**Overall importance**
**Session**							
*User*	✓	✓	✓	✓	✓	✓	★★
*(User Credentials)*							
*Start_date*			✓			✓	★
*(Session start date)*							
*End_date*			✓			✓	★
*(Session end date)*							
*Computer_ip*					✓	✓	★
*(computer IP)*							
*User position/Role*				✓			○
***Event***							
*Date/Hour*	✓	✓	✓	✓	✓	✓	★★
*(Event date)*							
*Patient_ID*			✓	✓	✓	✓	★★
*(Patient Identification)*							
*Report_ID/ Document_ID /Information Accessed*		✓	✓	✓	✓	✓	★★
*(Information accessed)*							
*Patient Location (Ward)*			✓	✓			★
*Actualization Reason/ Reason for Update*			✓		✓		★
*Chart Access Reason*				✓			○
*Document Code*			✓				○
*Document Type*			✓				○
*Event_Description*				✓		✓	○
*(Event description)*							
*Orders Entered*				✓			○
*Service/Department*				✓			○
*Session_ID*						✓	○
*(Session Identification)*							
*User’s Place of Work (Ward)*			✓				○
*Source of Access*					✓		○
*Outcome indicator*	✓						○
*Event_Id*	✓						○
*Participants_Object_Id*	✓						○

LEGEND: ✓ – mandatory in study/report of each column; ★★ – essential; ★– important; ○ optional.

The ISO 25012 [30] was also used to define the dimensions of quality of data applicable to AT.

### Processing the collected AT

**Figure 1:**
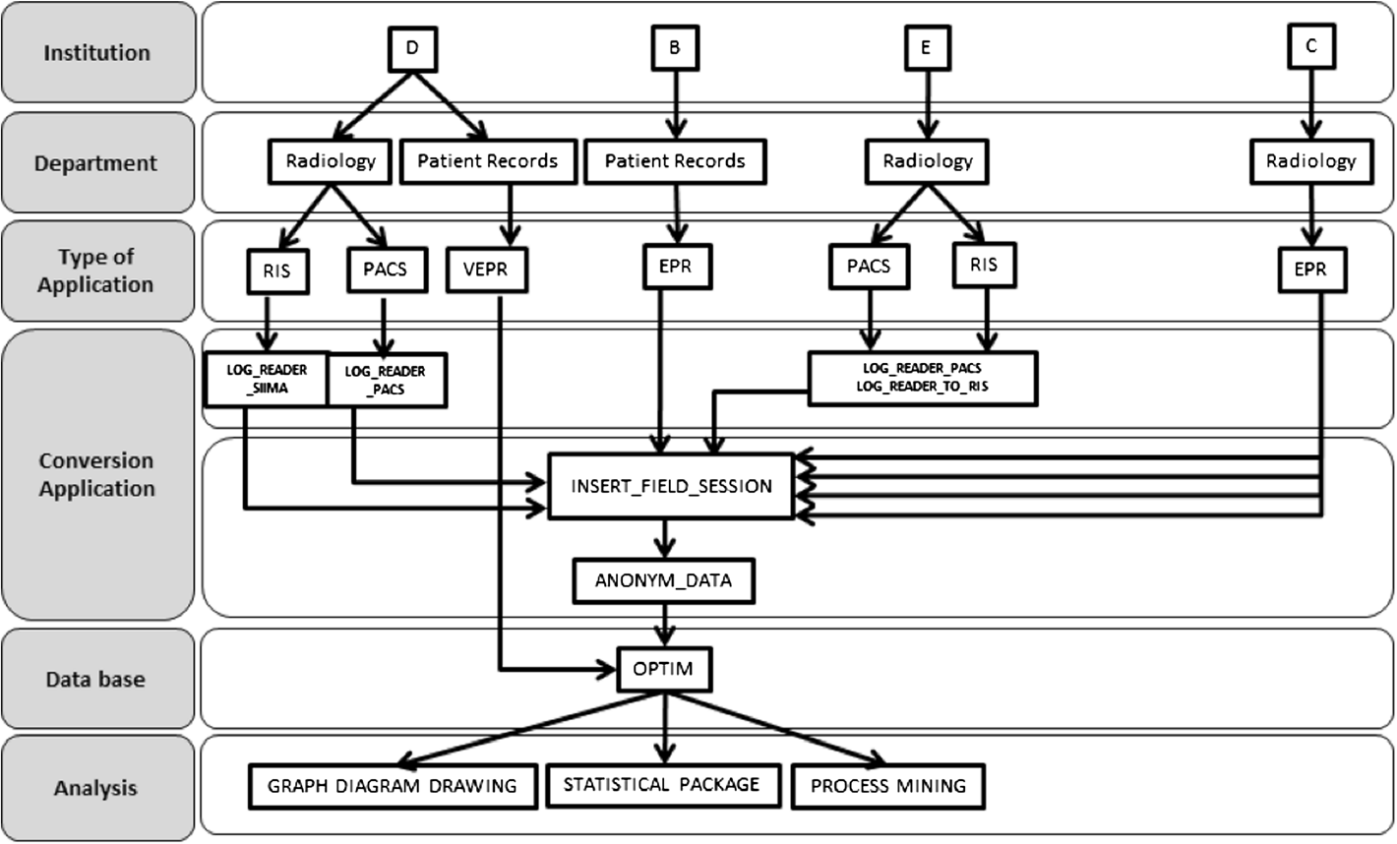
**AT collected and treated.** RIS: Radiology Information System; PACS: Picture Archiving and Communication System; VEPR: Virtual Electronic Patient Record; EPR: Electronic Patient Record.

It was necessary to process/analyse the collected AT. For this, some computer applications were created: 1) one of these applications removed data not relevant to our study (e.g. numeric values that had no meaning for us), 2) another application created a new field (COMPUTER_SESSION) when it is not present (generated by using other fields like USER, START_DATE and END_DATE), 3) and an application for the anonymization of user and patient identification (see Figure 1).

In Figure 1, the first row (Institution) represents the 4 hospitals from which AT data were collected; the second row (Department) represents the department that contains the application from which the AT were extracted; the third row (Type of Application) represents the type of application in each department from which AT were extracted; the fourth row (Conversion Application) represents the computer applications created to 1) remove data not relevant to our study, 2) insert the session field and 3) anonymize user and patient identification; the fifth row (Database) represents the database where every information collected from hospitals was saved; the sixth row (Analysis) represents the software used to analyse data [31].

The major difficulties encountered during this stage were:

•Data received from different institutions and different type/specialties were not always comparable (e.g. institution B sent information about Patient Record and institution E sent information about Radiology).

•Missing data (e.g. computer_ip, session start_date, session end_date).

•The format used in some AT is very confusing: no characters to separate values, some events had multiple lines and some AT were distributed by several files due to balanced servers.

### Assessing the quality of AT

The quality of existing AT in the Portuguese Hospitals was assessed using (a) the previously defined most important data elements to include in an AT (see section “Data elements important for the completeness of AT”), and (b) the quality dimensions applicable to AT.

### Ethical approval

The Health Ethical Commission of the HSJ approved this study (Comissão de Ética de Saúde do HSJ), having the reference number 45/2010.

## Results

### Opinions of hospital CIOs regarding AT

The main questions and answers obtained from the interviewees are presented in Table 3. These interviewees were only certain of one IS allowing AT in each of their institutions (in average there were identified 21 different IS within these hospitals [32]). At least one more IS with AT is known by the authors to exist in 4 of the 5 hospitals, although the representatives did not knew about them. In only one of these hospitals the AT feature was mentioned in the requirements for the development and implementation of new IS. In one of the hospitals there was even a Laboratory Information System (LIS) that although it had the ability to maintain AT, the IT department staff decided to disabled it.

**Table 3:** Questions made to IT department representatives of hospitals about AT in their IS

**Question**	**Answers**
What is the number of IS that have AT?	• One IS per institution. Actual answers were: LIS (Laboratory Information System) and PACS (Picture Archiving and Communication System) (twice). There are computer applications that, although with the ability to store AT viewing, have this functionality disabled (e.g. Pathology Lab IS)
What is the frequency that someone asks to use this data?	• All representatives said very rarely or none. One representative argued that very few people knew that it was even possible to have this information
What were the reasons to access AT?	• Only one representative answered that the AT were used (very rarely) to audit if doctors have seen the radiology reports in ER before making the patient discharge
What are the potential main benefits to record these AT?	• It allows the institution to reason about the usefulness of each software component and calculate its cost-benefit
	• It helps to do health service research
	• It may legally support medical decisions
	• It may dissuade inappropriate user access to patient data
What are the main problems to record those AT?	• Too much data to maintain (one answer)
	• Makes the systems slower (one answer)
Have you direct access to them, or need to ask the SW providers?	• Direct access, by accessing the database tables (all answered the same)

Regarding the frequency of access to the AT, all representatives mentioned that they accessed or were asked to access the AT very rarely or never. They did not feel it was their duty to control who was accessing patient data. It was also mentioned that very few people outside the IT departments knew it was even possible to collect this data, and that if other people knew maybe they would ask for it.

Concerning the reasons to access AT, one representative said that he, together with the responsible of the radiology department used them to audit which doctors were discharging patients from the Emergency Room without viewing the reports. The other three representatives did not remember of ever using them.

Four potential benefits were stated in the interviews. One representative intended to use AT to maintain or to remove IS implemented functionalities based on the amount of usage they had. Another mentioned that further health service research could be performed (e.g. workflows) based in these AT. Checking what patient data was available and was seen by health professionals at the time of clinical decisions for legal or ethical reasons was also stated. Finally, it was mentioned that the announcement of AT could dissuade inappropriate access to patient data.

The two referred problems associated with AT were the amount of disk space they took, and the fact that they would make the IS work slower. Regarding the access to the AT data, all representatives said they would need to access directly the IS database tables if they wanted to audit the access of a particular user to a piece of patient data.

### Data elements important for the completeness of AT

Three groups of importance were created according to the frequency of references to specific data elements (see column overall importance in Table 2):

– Essential. These were the data elements that were referenced more than 4 times (see Table 2). These were considered mandatory for a complete AT.

– “User”, “Event Date/Hour”, “Patient ID”, and “Report ID/Document ID/Information Accessed”.

– Important. These were the data elements that had 2 or 3 references.

– “Start Date”, “End Date”, “Computer IP number”, “Patient Location”, “Reason for Update” and “Event Description”.

– Optional. All the other fields were considered optional.

– “User position/Role”, “Chart Access Reason”, “Document Code”, “Document Type”, “Orders Entered”, “Service/Department”, “Session ID”, “User’s Place of Work”, “Source of Access”, “Outcome of event”, “Participants ID”, “Event ID”.

### Quality of data dimensions applicable to AT

Apart from the completeness of AT, other dimensions were also considered.

There are several general approaches to classify data quality problems (e.g. dirty data), with different definitions and interpretations. Among them we may find comprehensive enumerations of data quality problems [33-35], a definition of taxonomy for data quality anomalies in a database perspective [36], and a taxonomy focused on time-oriented data quality problems [37]. Research on data quality identified many different data desirable attributes (dimensions), but there is not a total agreement on their definitions. For instance, Weiskopf and Weng [38] the literature and identified five common dimensions (completeness, correctness, concordance, plausibility, and currency) for the assessment of data quality in the context of electronic health record data reuse for research, and found a great variability and overlap in the terms used to describe each dimension. We feel that the option for the generic data quality model proposed by the ISO 25012 [30] standard is important to guarantee a domain independent perspective.

The ISO 25012 is a standard composed of 15 generic quality dimensions. Although this standard addresses data quality in software engineering, we believe that it could also be applied to AT data with a few adaptations. These adaptations consisted in removing dimensions that we felt that were part of AT intrinsic definition (Currentness) or that were not applicable (Confidentiality, Efficiency and Portability).

These dimensions need to be instantiated into more practical factors to be assessed. These factors were defined based on our previous experience and on quality issues found in the collected AT. Table 4 presents the dimensions addressed by ISO 25012 (e.g. “1. Completeness”, “2. Consistency”), and the practical factors we analysed in each of these dimensions (e.g. “1.1 Percentage of important fields to AT”, “2.1 Actions performed after logout”).

**Table 4:** Results of ISO 25012 standard analysis

	**Dimension**	**Institutions**						
		**D**			**C**	**B**	**E**	
		**RIS**	**PACS**	**VEPR**	**EPR**	**EPR**	**PACS**	**RIS**
**1**	**Completeness**							
**1.1**	Percentage of existing essential fields	100%	50%	100%	100%	50%	75%	50%
**1.2**	Completeness (i.e. without missing values) of sent data	66%	20%	40%	96%	34%	32%	31%
**2**	**Consistency**							
**2.1**	Percentage of actions after logout	---	---	1.6%	---	---	---	---
**2.2**	Percentage of fields filled differently in similar situations	NA	NA	4.34%	NA	NA	14.2%	NA
**3**	**Comprehensibility**							
**3.1**	Is the information structured?	NO	NO	YES	YES	YES	NO	NO
**3.2**	Number of fields with intuitive name /Number of fields	46%	46%	83%	83%	100%	64%	46%
**4**	**Traceability**							
**4.1**	Is there a special functionality to know who accessed logs?	NO	NO	NO	NO	NO	NO	NO
**5**	**Accessibility**							
**5.1**	There is a restricted access to logs with user and password?	YES	YES	YES	YES	YES	YES	YES
**5.2**	Are logs easy to query?	NO	NO	YES	YES	YES	NO	NO
**6**	**Credibility**							
**6.1**	Duration of Sessions (average)	---	---	10 m^1^	---	---	---	---
**6.2**	Is there a user profile?	---	---	YES	---	YES	---	---
**7**	**Accuracy**							
**7.1**	Is the DATE format coherent?	YES	YES	YES	NO	YES	---	YES
**7.2**	Can we distinguish the time zone? (Summer/Winter)	NO	NO	NO	NO	NO	NO	NO
**8**	**Precision**							
**8.2**	Are the seconds of the actions recorded?	YES	YES	YES	SOMETIMES	YES	YES	YES
**8.3**	Are the milliseconds of the actions recorded?	NO	NO	NO	NO	YES	NO	NO
**9**	**Recoverability**							
**9.1**	Are there backups?	---	---	YES	---	---	---	---

1 – When the timeout happens; *NA*: Not applicable, – Unknown.

The process mining researchers are also starting to give special attention to the quality of AT. In Mans et al. and Bose et al., several real-life event logs (some related to healthcare) are analysed to illustrate the existence of process and event log problems/issues [39,40]. The authors also expect encourage systematic logging approaches, repair techniques and analysis techniques.

### AT collected and their quality

Every institution was contacted by phone and emailed in the beginning of 2011. In Hospital B, AT were collected from the Electronic Patient Record (EPR); in Hospital C they were collected from the use at Radiology data in an EPR; in Hospital D they were collected from Radiology Information System (RIS) and Picture Archiving and Communication System (PACS), and Patient Record from Virtual Electronic Patient Record (VEPR); and in Hospital E they were collected from RIS and PACS.

### Completeness

Complete information is sufficient in depth, breadth, and scope to be used as an audit trail.

Only 3 out of the 7 collected AT included the 4 essential fields in their structure (see Table 4). The AT provided had from 13 to 69 data fields (26 in average). Some AT had many missing values in these data fields. None of the AT were satisfactory, and some were very poor (e.g. PACS in Hospital D).

### Consistency

Consistent information is free from contradiction and coherent with other information.

The dimension consistency was analysed by exploring actions in applications that we knew did not make sense regarding the natural flow of application usage, and situations that were similar although recorded with different descriptions.

Unfortunately in only one AT (from VEPR of Institution D), was there enough information (number of cases and variables) for us to analyse. This analysis was performed by: (1) modelling user behaviour by designing graphs that illustrate the sequence of actions performed by users and (2) finding strange patterns (e.g. people doing the same action repeatedly in very small intervals of time, or just searching for patients without actually seeing any information about them or performing actions without a login). In the analysed AT a significant number of inconsistent cases occurred (in 1.6% of all sessions logouts there is a performed action after the logout).

In two systems of the same Institution E there were semantic issues related to recording the same actions with different descriptions (4.34% in VEPR and 14.2% PACS). These cases are probably due to using the same log files for updated software versions that describe similar actions with different names (e.g. logout and sign-out).”

### Comprehensibility

Comprehensible information has attributes that enable it to be read and interpreted, are expressed in appropriate languages, symbols and units.

The dimension comprehensibility was sub-divided in having the AT structured and having the different fields named in an intuitive way. Only 3 of the 7 received AT have a proper structure (the other 4 were not in a tabular format and seemed to be mainly debug logs). And, in these 3, the naming of the fields was not descriptive of the contents, making it very difficult to understand their meaning.

### Traceability

Tracable information includes owner or author of the information, and any changes made to the information can be checked.

Regarding traceability of accesses to the AT, none of the solutions had a special functionality to prevent and record accesses to the AT. This had to be performed based on the Operating System log files of the servers holding the AT.

### Accessibility

Acessible information is able to be processed and read.

Accessibility was divided into controlling the access to the AT and the easiness to process the data in them. As stated in traceability the access was controlled by Operating Systems’ authentication control in PACS and RIS and by Database authentication control in the EPR or VEPR.

### Credibility

Credible information is reputable, unbiased / objective, and trustable / believable.

This item aims to evaluate if one can trust the user’s identification associated with each action that is recorded in the AT. This aim was sub-divided into (1) checking if the duration of user sessions was too long (which indicates that there was no session timeouts and therefore session was shared among users) and (2) the inexistence of user profiles in the AT. Only one system (VEPR) included information regarding session timeout, and in only two of other systems there was a user role in the AT.

### Accuracy

Accurate information has a correct representation of the true value of the intended attributes of a concept.

Regarding accuracy, special attention was given to date information, namely the coherence of date information throughout the AT and the distinction of time zones and Summer/Winter time. Regarding the coherence, in one of the AT dates with different formats were found, and none of the formats included time zones (not so important) or Summer/Winter time (much more important).

### Precision

Precise information provides attributes that are exact or enough discrimination.

Regarding precision, special attention was given to dates. Only one system recorded the milliseconds of actions (EPR at B), another system did not record the seconds in all the events (EPR at C), and all the others recorded the seconds but not the milliseconds.

### Availability

Available information can be retrieved by authorized users and/or applications.

The availability dimension was not evaluated, as these AT were the ones made available to us by the Hospitals. Nevertheless, it is important to state that Hospitals had difficulties to collect some of these AT which were only solved with the intervention of the software suppliers.

### Recoverability

Recoverability is related to maintaining and preserving a specified level of operations and quality, even in the event of failure.

Regarding recoverability, in only one of the Hospitals it was possible for us to confirm the existence of backups regarding their AT.

## Discussion

### Global

Although there is some awareness for the need to have quality in AT, the existing ones are very poor and not able to provide suitable traceability or help analyse HIS usage.

Most of the concerns found are not related to key technical difficulties, but probably more related to poor understanding of what should be present on an AT and not giving enough importance to record the actions of users in HIS. In our opinion, the source of this problem is associated with the fact that customers (health institutions) do not request software capable of delivering proper AT from their software providers.

### Opinions of Hospital CIOs regarding AT

It was obvious, from the interviews that there was little concern in confirming if AT existed, were being maintained and properly secured. Also, little concern in including this feature as requirements for new IS or upgrades.

### Data elements to include in AT

We argue that the data elements qualified as essential should be made mandatory for AT in any IS acquired. In particular, the correct identification of patient and user is crucial. On the other hand, the identification of the reports / documents is more difficult, as no document nomenclature has widespread use. The RFC 3881 [41] or IHE-ATNA provides a good starting point and has been used in several projects and regulation as epSOS or more recently “Meaningful Use Stage 2 Proposed Rule”.

One of the most important pieces of AT is the time when each action is performed. For it to be comprehensible, the values must have sufficient detail (seconds or milliseconds) and be unambiguous. The difficult part to guarantee is unambiguity, due to differences in storing time formats, to un-synchronization of clocks and to changes in summer time (i.e. daylight saving time). We recommend dates to be stored as complete dates [42] and that all servers have their clocks synchronized with each other using known standards [43] and with an official time server.

To improve the session information, it can be useful that, besides username, start and end session date, the terminal IP number used to access information and how was the session terminated (e.g. user, timeout or other) is also stored. The IP number can be used to more effectively re-create scenarios of information access, and to detect irregular behaviours as having the same users logged in two different terminals at the same time. The method used to terminate the session can be used to audit different user habits about letting the session opened for other users to use the IS.

Apart from session start and end, the AT should also record patient searches, data changes and the visualization of patient data. Although the recording of data insert and updates is more common, recording patient searches and visualization of patient data can help attain some of the most important potentialities of the AT, namely for health service research and to legally support medical decisions.

### Quality of data dimensions to use in AT

Another requirement, that is seldom observed, is that the simple access to the AT, including the audit control functions and the audit files, should always be controlled to ensure the integrity of the records. The implications of security in AT are vast and can be harmful.

Health information managers must confirm that the auditing functions are turned on and fully functional. In fact, health information managers would be quite surprised to find that AT systems are not active or their resulting audit files are kept only for a rather small period of time.

### Analysis of the existing AT in Portuguese hospitals

The AT was analysed according to the standard ISO 25012. To improve the quality of logs, we suggest 1) the existence of a well-defined structure for readability; 2) that every field is completed and 3) that the fields always use the same format. Not using a standard to record audit trails represents a major drawback when analysing data and therefore makes it very difficult to use AT to improve HIS. The use of ISO 25012, ISO/TS 18308:2004, ISO/IEC 27001:2006, XES Standard [44] and IHE-ATNA should be used to define requirements. We also recommend performing a periodical audit access to all data of a random sample of patients.

The analysed audit trails were very poor regarding completeness. Many essential or important data elements were missing making the interpretation of what the users actually did very hard. This issue also made the consistency dimension difficult to analyse in most of the AT. Nevertheless, in the only one with enough data, actions inconsistent with the proper use of an HIS were found.

The traceability of access to the AT was completely missing in the studied cases and the accessibility was controlled by the Operating System or DBMS. The ISO/TS 18308:2004 and ISO/IEC 27001:2006 standards and the IHE-ATNA regulation or similar regarding controlling access to the AT should be seriously considered if the AT is to be used in a legal environment, namely as a proof that someone has accessed or not a particular patient record.

Regarding credibility, not all of the important events (e.g. session logout) are recorded in the AT.

Regarding accuracy and precision the main issues are related to date and time. The formats found were inconsistent and not with enough precision. No AT used the ISO 8601 format. This situation is also a major drawback to the safe use of this data.

Finally, we also advocate the existence of an auditing visualization tool that could easily present 1) all actions performed by an user, 2) all actions performed on the record of a patient, and 3) the navigation flows of groups of users by presenting them on graphs and using graph theory to analyse them.

## Conclusions

Although there is some awareness for the need to have quality in audit trails, the existing AT are very poor and not able to provide proper traceability or help analyse Health Information Systems usage. Regarding the AT analysed, the lack of internal structure, data quality and precision limits it’s useful for legal issues and health information systems improvement. Existing standards (e.g. ASTM: E2147, ISO/TS 18308:2004, ISO/IEC 27001:2006) are still not broadly used in Portugal.

## Competing interests

The authors declared that they have no competing interests.

## Authors’ contributions

RCC was responsible for the study design, organization, interviews and final manuscript writing. IB was responsible for the Audit trails processing and initial manuscript preparation. CPE, LL, PPR, AF and RCC advised the study design, and supervised the analysis, the results interpretation and the manuscript preparation. All authors read and approved the final manuscript.

## Pre-publication history

The pre-publication history for this paper can be accessed here:

http://www.biomedcentral.com/1472-6947/13/84/prepub
